# Strong increase of true and false positive mycobacterial cultures sent to the National Reference Centre in Belgium, 2007 to 2016

**DOI:** 10.2807/1560-7917.ES.2019.24.11.1800205

**Published:** 2019-03-14

**Authors:** Karine Soetaert, Lorenzo Subissi, Pieter-Jan Ceyssens, Brigitte Vanfleteren, Marianne Chantrenne, Tommi Asikainen, Els Duysburgh, Vanessa Mathys

**Affiliations:** 1 Unit Bacterial Diseases Service, Infectious diseases in Humans, Sciensano, Brussels, Belgium; 2 European Public Health Microbiology Training Programme (EUPHEM), European Centre for Disease Prevention and Control, Stockholm, Sweden; 3 Laboratory of medical microbiology, Sciensano, Brussels, Belgium; 4 Epidemiology of Infectious Diseases, Sciensano, Brussels, Belgium; 5 Healthcare-associated infections and antimicrobial resistance, Sciensano, Brussels, Belgium

**Keywords:** mycobacteria, nontuberculous, species identification, Belgium, bacterial infections, tuberculosis, Mycobacteriacea, public health policy, surveillance, laboratory surveillance, epidemiology, laboratory

## Abstract

**Introduction:**

In 2007, a new federal legislation in Belgium prohibited non-biosafety level 3 laboratories to process culture tubes suspected of containing mycobacteria.

**Aim:**

To present mycobacterial surveillance/diagnosis data from the Belgian National Reference Centre for mycobacteria (NRC) from 2007 to 2016.

**Methods:**

This retrospective observational study investigated the numbers of analyses at the NRC and false positive cultures (interpreted as containing mycobacteria at referring clinical laboratories, but with no mycobacterial DNA detected by PCR in the NRC). We reviewed mycobacterial species identified and assessed trends over time of proportions of nontuberculous mycobacteria (NTM) vs *Mycobacterium tuberculosis* complex (MTBc), and false positive cultures vs NTM.

**Results:**

From 2007 to 2016, analyses requests to the NRC doubled from 12.6 to 25.3 per 100,000 inhabitants. A small but significant increase occurred in NTM vs MTBc proportions, from 57.9% (587/1,014) to 60.3% (867/1,437) (p < 0.001). Although NTM infection notification is not mandatory in Belgium, we annually received up to 8.6 NTM per 100,000 inhabitants. *M. avium* predominated (ca 20% of NTM cultures), but *M. intracellulare* culture numbers rose significantly, from 13.0% (74/587) of NTM cultures in 2007 to 21.0% (178/867) in 2016 (RR: 1.05; 95% CI: 1.03–1.07). The number of false positive cultures also increased, reaching 43.3% (1,097/2,534) of all samples in 2016.

**Conclusion:**

We recommend inclusion of NTM in sentinel programmes. The large increase of false positive cultures is hypothesised to result from processing issues prior to arrival at the NRC, highlighting the importance of sample decontamination/transport and equipment calibration in peripheral laboratories.

## Introduction

The highly diverse group of nontuberculous mycobacteria (NTM) encompasses all mycobacterial species except the *Mycobacterium tuberculosis* complex (MTBc) and *M. leprae*. In contrast to the obligate pathogen nature of the MTBc, which is the causative agent for tuberculosis (TB), NTM are environmental bacteria, which are increasingly being recognised as important opportunistic pathogens [1]. Although their most common clinical manifestation is respiratory disease, NTM are also commonly isolated from lymph nodes, skin and soft tissue, bone and joint infections [1]. Only a handful of species account for the majority of clinical cases, most notably those of the *M. avium* complex, *M. kansasii* and *M. abscessus* complex [2]. The correct identification of the aetiological agent is critical for diagnosis and patient management, as species differ in clinical relevance, antibiotic susceptibility and treatment outcome [3].

For more than two decades, the global disease burden of TB has been monitored under close supervision of the World Health Organization (WHO) [4]. In sharp contrast, obtaining accurate epidemiological estimates of the infectious burden of NTM remains challenging as these infections are not routinely reported to public health authorities. Regardless of this constraint, and in contrast to yearly declines in both TB mortality rate and incidence, recent reports indicate increasing numbers of NTM disease and colonisation in high-income countries [5-7]. The reasons are unclear, but often attributed to improved culturing technique, increased awareness, and actual increase in disease incidence [8]. Interestingly, this global increase was recently contradicted by a 25-years (1991–2015) retrospective survey in Denmark [9] showing no increase in NTM disease incidence or proportion of patients with positive NTM cultures. This highlights the need for systematic approaches for diagnosing and surveillance of NTM.

In Belgium, mycobacterial species identification is routinely performed in 12 biosafety Level 3 (BSL3) clinical laboratories and in the National Reference Centre for mycobacteria (NRC). Clinical laboratories without BSL3 can send cultures suspected of containing mycobacteria either to one of the BSL3 laboratories or directly to the NRC. When species of the MTBc are identified, the BSL3 laboratories, or the NRC, perform first-line drug susceptibility testing (DST). Notifications of active TB disease to health authorities is mandatory, and BSL3 laboratories only send physical strains to the NRC in case of need for second-line DST. In contrast, NTM isolates are not actively monitored, and cultures are sent voluntarily to the NRC for species confirmation and DST. While few BSL3 laboratories have the technique available in Belgium to conduct DST on NTM species, the vast majority (> 95%) of such tests are performed at the NRC by broth microdilution and Canetti methods [10]. The NRC performs a DST for all clinically-relevant NTM complying with the American Thoracic Society/Infectious Disease Society of America (ATS/IDSA) criteria [11]. The compliance with these criteria are based on clinical, radiological and microbiological evaluation performed by the requesting clinician.

In 2007, the federal government introduced a legislation stating that a culture tube suspected for presence of mycobacteria (i.e. flagged positive), cannot be opened unless confined in a biological safety cabinet inside a BSL3 area. This culture can either have been flagged positive by automated systems monitoring mycobacteria growth indicator tubes (MGIT), or by a trained laboratory worker, based on colony morphology on mycobacterial selective agar. Although the legislation dates from 2007 it was only gradually implemented in the field with the consequence that more and more cultures were sent to the NRC or other BSL3 laboratories for species confirmation. 

In this work, we present an overview of mycobacterial surveillance in the past decade and diagnosis at the Belgian NRC. We describe trends in mycobacterial species, evolution in sample turnover, false positive cultures (interpreted as containing mycobacteria at the referring clinical laboratory, but with no mycobacterial DNA detected by PCR in the NRC) and type of requested analyses, and also present recommendations towards peripheral laboratories and Belgian government bodies for improved surveillance.

## Methods

### Clinical isolates

This study comprises all suspected positive cultures received from 1 January 2007 to 31 December 2016, by the Belgian NRC for species identification and/or DST. These cultures were sent from 107 different Belgian clinical laboratories from all over the country.

### Species identification

After reception of suspected positive cultures, identification of MTBc was performed by PCR detection of the Insertion Sequence IS*6110*, only present in the genome of the MTBc members [12]. In case of negative IS*6110* PCR, identification of possible NTM species was initiated by Sanger sequencing of the 16SrRNA gene [13]. The obtained sequences were subjected to Basic Local Alignment Search Tool (BLAST) analysis against the National Centre for Biotechnology Information (NCBI) sequence databases and the Ribosomal Database Project (RDP) database [14]. In case of non-conclusive BLAST result, or suspicion of mixed infections, other molecular assays were used such as the commercial GenoType CM/AS Line Probe Assay (Hain Lifescience, Nehren, Germany) based on the analysis of the 23SrRNA gene or in-house species-specific PCRs.

### Statistical analysis

We used STATA 13.0 for statistical analyses. Pearson’s chi-squared test was used to compare culture results according to (i) the medium used and (ii) the sample type (pulmonary vs non-pulmonary/unknown). The Cochran–Armitage test for trend was applied to study the trend of positive cultures (proportion of MTBc vs NTM over the study period). We applied a Poisson and negative binomial regression to explore trends in the proportion of false positives and NMT species by using relative risk (RR). Cost calculations for negative sample processing were based on the price of consumables necessary to perform the tests to exclude presence of mycobacteria.

### Ethical statement

All data recorded in the context of the present study were not collected for research purposes but as part of the routine data collection for diagnosis. Consequently, no ethical approval was required. Anonymity of data was ensured before analysis.

## Results

### Increasing sample numbers at Belgium’s National Reference Centre for mycobacteria

From 2007 to 2016, the NRC for mycobacteria in Belgium characterised a total of 18,011 cultures sent by 107 laboratories. Among the total number of cultures, 75.9% (13,678/18,011) were isolated from respiratory specimens including sputum, bronchoalveolar lavage, bronchial aspiration etc.. Overall, a total of 7,433 (41.3%) strains were identified as NTM, 4,885 (27.1%) belonged to the MTBc, and 5,693 cultures (31.6%) tested negative for mycobacteria. The number of annually received cultures doubled from 1,265 in 2007 to 2,534 in 2016 (Table), corresponding to an increase of 12.6 to 25.3 per 100,000 inhabitants. Although the majority of the additional samples were false positive samples (see below), we observed a substantial increase in the absolute number of NTM (from 587 to 867) and MTBc (from 427 to 570) isolates, with the ratio NTM/MTBc increasing significantly from 57.9% (587/1,014) (2007) to 60.3% (867/1,437) (2016) (p < 0.001).

**Table:** Absolute numbers of samples received by the National Reference Centre for mycobacteria and proportions of different sample types, Belgium, 2007–2016 (n = 18,011)

Year (total number of isolates analysed)	MTBc		NTM		False positive^a^	
	n	%	n	%	n	%
2007 (1,265)	427	33.8	587	46.4	251	19.8
2008 (1,388)	479	34.5	564	40.6	345	24.9
2009 (1,462)	409	28.0	698	47.7	355	24.3
2010 (1,517)	517	34.1	672	44.3	328	21.6
2011 (1,765)	493	27.9	696	39.4	576	32.6
2012 (1,743)	439	25.2	715	41.0	589	33.8
2013 (2,042)	539	26.4	881	43.1	622	33.8
2014 (1,929)	460	23.9	857	44.4	612	31.7
2015 (2,366)	552	23.3	896	37.9	918	38.8
2016 (2,534)	570	22.5	867	34.2	1,097	43.3
**Total (18,011)**	**4,885**	**27.0**	**7,433**	**41.3**	**5,693**	**31.6**

MTBc: *Mycobacterium tuberculosis* complex; NTM: nontuberculous mycobacteria.

^a ^Interpreted as containing mycobacteria at the referring clinical laboratory, but with no mycobacterial DNA detected by PCR in the Belgian National Reference Centre for mycobacteria.

### Substantial increase in the proportion of false positive cultures

During the last decade, we witnessed a steep increase in the proportion of cultures flagged positive at the referring clinical laboratory but in which no mycobacteria genetic material detected by PCR in the NRC (false positive cultures). From 22.7% (1,279/5,632) of the total sample number for the period 2007–10, this proportion increased to 32.1% (2,399/7,479) between 2011 and 2014, a period where the legal obligation to send a positive mycobacterial culture to a BSL3 laboratory was reinforced in the field. The observed increase became even more dramatic in the last 2 years of the study period (41.2% (2,015/4,900), p < 0.001, Table), with 43.3% (1,097/2,534) of all mycobacterial cultures being false positives in 2016. Comparing years 2007 and 2016, this increase is statistically significant (RR: 2.18; 95% confidence interval (CI): 1.90–2.50; Supplement Table S1). Notably, negative sample processing includes inoculation, immunochromatography, DNA extraction and three consecutive PCRs, as part of the routine diagnosis workflow to be sure a weakly positive sample is not missed. This adds up to 65 EUR in consumable cost per sample, totalling up to 370,045 EUR for the entire study period.

When considering the type of growth medium used for primary mycobacterial culture in peripheral laboratories in terms of false positivity, it was observed that liquid BacT/ALERT and MGIT media gave the highest proportion of false positive samples (40.2% (2,444/6,077) and 31.7% (2,844/8,967), respectively), as compared with solid medium (10.1% (190/1,872), p < 0.001, Supplement Table S3). The proportion of false positive samples did not change by sample type (Supplement Table S2).

### Proportions and trends for mycobacterial species

The NRC performs (sub)species identification on all mycobacterial cultures (Supplement Table S4). Within the MTBc, 96% (4,669/4,885) of the samples were *M. tuberculosis*, with very few *M. africanum*, *M. bovis* and *M. bovis* bacillus Calmette–Guérin (BCG) strains each year (Supplement Table S4). The ten most prevalent NTM species represent > 95% of all NTM species in Belgium (Figure; Supplement Table S4). Between 2006 and 2017, ca 20% (1,472/7,433) of the NTM positive cultures were *M. avium*. We did not find a statistically significant trend in the proportion of positive *M. avium* cultures. Comparing 2007 to 2016, the proportion of *M. intracellulare* complex among all NTM positive tests increased from 13.0% (74/587) to 21.0% (178/867), and this increase was statistically significant (RR: 1.05, 95% CI: 1.03–1.07). For the same 10-year period, the proportion of *M. xenopi* among all NTM positive tests decreased significantly from 21.0% (126/587) to 15.0% (127/867) (RR: 0.69; 95% CI: 0.54–0.88). The observed increase of the proportion of *M. abscessus-chelonae* complex from 3.6% (21/587) to 5.2% (45/867) was borderline significant (RR: 1.04; 95%CI: 1.00–1.07), while that of the environmental contaminant *M. gordonae* from 21.0% (121/587) to 23.0% (198/867) was not significant (RR: 1.12; 95% CI: 0.90–1.4).

**Figure:**
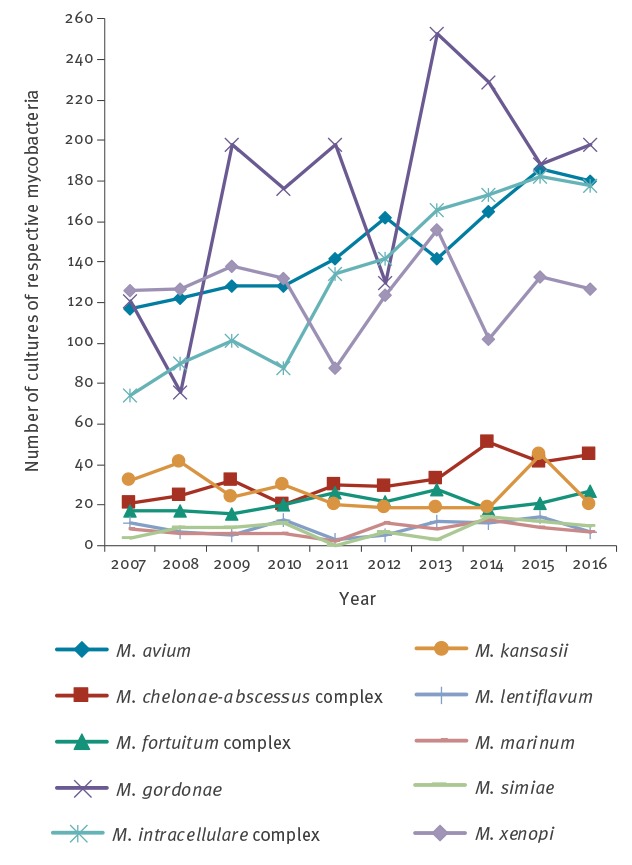
Incidence (absolute numbers) over time of the 10 most prevalent nontuberculous mycobacteria identified from mycobacteria-positive cultures at the National Reference Centre for mycobacteria, Belgium, 2007–2016 (n = 7,433)

We also registered increasing DST on NTM species, which is only performed upon physicians’ request, from 28% (165/587) in 2007 to 35% (305/867) in 2016. Comparing 2007 to 2016, the proportion of requested DST on NTM among all NTM positive cultures increased significantly (RR: 1.25; 95% CI: 1.04–1.51).

## Discussion

Two main outcomes come forward from this retrospective observational study, which presents the analysis of sample turnover at the Belgian NRC for mycobacteria during the last decade (2007–16). First, while the numbers of samples for all confirmed mycobacteria rose, the proportion of NTM cultures increased only slightly over the study period. A second observation was the rapidly increasing number of false positive cultures sent by peripheral laboratories.

Historically, public health surveillance in the area of mycobacteria is strongly focused on members of the MTBc. In many high-income countries this situation is changing as infections with NTM are increasingly reported. In Belgium, these represented up to 8.6 of 100,000 inhabitants. In the last decade, the importance of some species groups has clearly increased, like the *M. intracellulare* complex with the most predominant subspecies being *M. chimaera*. This pathogen has been implicated in severe cardiovascular and systemic infections following cardiac surgery through the use of contaminated ‘heater cooler’ devices [15,16], and accounted for 63% of the Belgian *M. intracellulare* isolates in 2015 [17].

In assessing the clinical importance of the results of the current study, we have to consider that increasing NRC sample numbers do not necessarily reflect increasing infection rates. Indeed, there are inherent difficulties in differentiating between disease, colonisation and contamination for NTM due to the ubiquitous character of these organisms [11]. Moreover, physicians are increasingly aware of the pathogenicity of NTM bacteria, reflected by significant increases in request for NTM drug resistance testing and a small but significant increase in the proportion of NTM vs MTBc, from 57.9% in 2007 to 60.3% in 2016 (Supplement Figure S1). As the notification of NTM infections is not mandatory in Belgium, more insights in clinical burden could be gathered by including NTM species in existing sentinel laboratory surveillance programmes.

The increasing number of false positive cultures sent by peripheral laboratories puts the current NRC workflow under greater pressure, as false positive cultures generate unnecessary laboratory work [18]. It seems plausible that the national policy, which prohibited the manipulation of any culture susceptible of containing mycobacteria outside a BSL3 might have had some impact on the number of false positive cultures. Indeed, clinical laboratories without BSL3 lost the opportunity to make a first sort, by microscopy, to detect contaminated and negative cultures. The legislation was introduced in 2007, but was only gradually implemented in the field. However, it is unlikely that the recent (2015–16) increase of false positive samples is directly connected to the wider policy application. A possible contributing factor might also be the switch to more automated detection of growth in liquid cultures, instead of visual inspection of colony morphology on solid medium, which more reliably excludes the presence of mycobacteria in the clinical sample.

In this respect, to limit the possibility of false positivity, we recommend laboratories to review sampling and sample decontamination procedures. Culture contamination rates of 3–5% on solid media or 5–10% in liquid media are acceptable [19]. If these limits are exceeded, laboratories should check that their decontamination procedure conforms to recommendations, and that it is properly performed [19]. When transport to the laboratory exceeds 1 hour, specimens for mycobacteriology (except blood) should be stored at 4 °C. Samples not available in duplicate should be kept at 4 °C after inoculation for general bacteriology until processing for mycobacteriology. The delay between collection and inoculation should not exceed 7 days [19]. We also encourage clinical laboratories to check the regular maintenance/calibration of their machines. 

There are nevertheless limitations of our observational study. The main one entails the lack of knowledge on sample preparation and shipment prior to arrival at the NRC. Indeed, external factors affecting sample quality were not investigated, such as data on whether different rates of contaminations occurred according to different submitting laboratories, data on transportation delays and cold chain maintenance. Moreover, as we defined false positive cultures as being devoid of mycobacterial DNA, we did not explore whether they were due to contamination. Finally, it is possible that various NTM isolates from the same patients were sent without specific notification and were counted separately. 

In conclusion, we report a slight increase in NTM among the mycobacterial cultures sent to NRC. Among these NTM, the most common clinically relevant species isolated were *M. avium*, *M. intracellulare* complex and *M. xenopi*. The analysis of the evolution of the species over the years suggests a steady increase of *M. intracellulare* complex infections, while the trends for the other NTM species did not follow a clear pattern. This study also reveals that a significant and increasing number of cultures sent to the NRC are false positives. A setup of the automated systems for mycobacteria growth or a revision of the decontamination/transport procedures by clinical laboratories might be suitable to avoid unnecessary subsequent laboratory work.

## Supplementary Data

173000 Supplement
