# Supplementary material for: Strong increase of true and false positive mycobacterial cultures sent to the National Reference Centre in Belgium, 2007 to 2016
Source: Euro Surveill. 2019 Mar 14;24(11):1800205. doi: 10.2807/1560-7917.ES.2019.24.11.1800205 (PMC6425549; doi:10.2807/1560-7917.ES.2019.24.11.1800205)
Supplement: Supplement [file 1800205_MATHYS_Supplement.pdf]

SUPPLEMENTARY MATERIAL

This supplementary material is hosted by Eurosurveillance as supporting information alongside the article "Strong increase of true and false positive mycobacterial cultures sent to National Reference Centre in Belgium, 2007 to 2016" on behalf of the authors who remain responsible for the accuracy and appropriateness of the content. The same standards for ethics, copyright, attributions and permissions as for the article apply. Eurosurveillance is not responsible for the maintenance of any links or email addresses provided therein.

Supplementary Figure 1 : Proportion NTM complex vs MTBc*

*Conchran-Armitage test for trend p<0.001

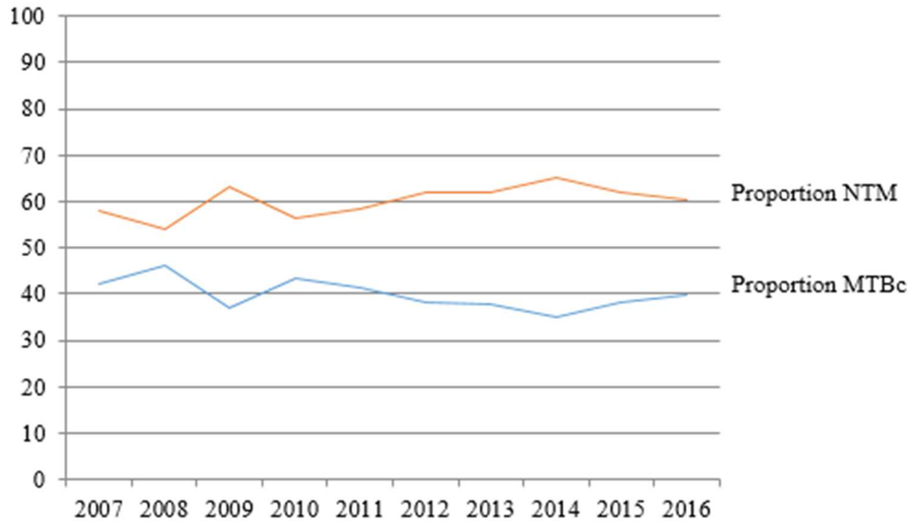

Supplementary Table 1: Relative risks (RR) for the association of false positive samples with time

Year	RR (95% CI)	p-value
2007	-	
2008	1.25 (1.06-1.47)	0.007
2009	1.22 (1.04-1.44)	0.014
2010	1.09 (0.92-1.28)	0.306
2011	1.64 (1.42-1.91)	0.000
2012	1.70 (1.47-1.97)	0.000
2013	1.54 (1.33-1.78)	0.000
2014	1.60 (1.38-1.85)	0.000
2015	1.96 (1.70-2.25)	0.000
2016	2.18 (1.90-2.50)	0.000

Supplementary Table 2: Percentage of false positive samples, NTM and MTBc by type of sample, 2007-2016.

	False positive, n (%)	NTM, n (%)	MTBC, n (%)	Chi^2 p-value
Type of sample				
Respiratory	4346 (31.7)	6063 (44.3)	3269 (23.9)	0.27
Non-respiratory/unknown	1356 (31.1)	1370 (41.1)	1616 (37.3)	

Supplementary Table 3: Percentage of false positive samples by type of culture medium used for first-line mycobacterial growth, 2007-2016.

Medium	False positive samples	Mycobacteria true positive samples	Total+	Chi^2 p-value
MGIT	2844 (31.7)	6123 (68.3)	8967 (49.8)	<0.001
BaCT/ALERT	2444 (40.2)	3633 (59.8)	6077 (33.7)	-
Bactec 9000	130 (14.3)	778 (85.7)	908 (5.0)	-
Solid medium	190 (10.1)	1682 (89.9)	1872 (10.3)	-

Row percentages are reported, unless specified.
+Column percentages

Supplementary Table 4: Species identification results for Non-tuberculosis mycobacteria from mycobacteria-positive cultures, 2007-2016.

	2007	2008	2009	2010	2011	2012	2013	2014	2015	2016
M. agri	1	0	0	0	0	0	0	0	0	0
M. alvei	0	0	1	0	0	0	0	1	0	0
M. anthracenicum	0	0	3	0	0	0	0	0	0	0
M. arupense	0	0	1	3	0	3	0	6	3	3
M. aubagnense	0	1	0	0	1	0	0	0	2	0
M. asiaticum	0	0	0	0	1	0	0	0	0	1
M. assiacum	0	0	0	0	0	0	0	1	0	0
M. avium	117	122	128	128	142	162	142	165	186	180
M. bohemicum	2	1	0	2	0	2	4	3	1	0
M. branderi	0	0	1	0	2	0	0	1	0	0
M. celatum	3	0	4	3	0	3	2	2	0	0
M. chelonae-abscessus cpx	21	25	32	20	30	29	33	51	41	45
M. chitae	0	0	0	0	0	0	0	0	1	0
M. colombiense	0	0	0	0	0	0	0	2	0	0
M. cookii	0	0	1	0	0	0	0	0	0	0
M. conspicuum	0	0	0	0	0	0	0	0	0	2
M. fluoranthenivorans	0	0	0	0	0	0	0	0	0	1
M. fortuitum cpx	17	17	16	20	26	22	28	18	21	27
M. frederiksbergense	0	0	1	0	0	0	0	0	0	0
M. genavense	2	0	0	1	2	0	0	0	0	0
M. gilvum	0	0	0	0	0	0	0	0	1	0
M. goodi	0	0	0	0	0	1	0	1	0	2
M. gordonae	121	76	198	176	198	130	253	229	188	198
M. haemophilum	2	2	0	0	2	0	0	0	0	0

M. heidelbergense	0	0	0	2	0	0	0	0	0	2
M. heckeshornense	2	0	0	0	0	3	1	0	0	0
M. heraklionense	0	0	0	0	0	0	0	1	4	0
M. holsaticum	1	1	0	1	0	0	1	0	0	0
M. hiberniae	0	0	0	0	1	0	0	1	1	0
M. immunogenum	0	0	0	0	0	0	0	0	2	0
M. interjectum	5	3	2	4	3	8	0	5	4	1
M. intermedium	2	2	5	3	0	1	0	0	0	0
M. intracellulare cpx	74	90	101	88	134	142	166	171	182	178
M. kansasii	32	41	24	30	20	19	19	19	45	20
M. komosense	0	0	0	0	0	0	0	0	1	0
M. kumamotonense	0	1	0	0	0	0	0	0	0	0
M. lentiflavum	11	7	5	13	3	5	12	11	14	7
M. llatzerense	0	0	0	0	0	0	0	0	1	1
M. mageritense	0	0	0	0	0	0	0	0	2	0
M. malmoense	9	7	3	9	8	7	7	8	10	8
M. mantenii	0	0	0	0	2	0	0	0	1	0
M. marinum	8	6	6	6	2	11	8	13	9	7
M. marseillense	0	0	0	0	0	0	0	0	1	1
M. massiliense	0	0	0	0	0	0	0	0	0	3
M. moriokanense	0	0	0	0	0	0	0	1	1	0
M. mucogenicum-ratisbonense cpx	0	0	3	2	3	2	5	2	2	8
M. nebraskense	0	0	0	0	0	1	0	1	1	2
M. non-chromogenicum	6	1	0	1	0	4	0	1	1	1
M. noviomagense	0	1	1	0	0	0	1	0	0	1
M. novocastrense	0	0	0	0	0	0	0	0	1	1
M. palustre	0	0	1	0	0	0	1	0	0	0

M. paraffinicum	8	0	2	0	0	0	0	0	0	0
M. parascrofulaceum	0	0	0	1	1	1	1	3	2	2
M. parmense	0	0	0	0	0	0	0	0	0	1
M. phlei	0	1	1	0	1	0	1	0	1	1
M. phocaicum	0	0	0	0	0	0	0	0	1	0
M. pulveris	0	0	0	0	0	1	0	0	0	0
M. saskatchewanense	0	0	0	0	0	0	0	0	2	2
M. scrofulaceum	0	6	1	0	3	4	6	2	0	1
M. senuense	0	0	0	0	0	0	0	0	1	0
M. setense	0	0	0	0	0	0	0	1	0	0
M. shagni	0	0	0	0	0	1	0	0	0	0
M. sherrisii	0	3	1	0	0	0	0	0	0	0
M. shimoidei	0	1	1	0	0	0	0	0	0	0
M. simiae	4	9	9	11	0	7	3	14	12	10
M. smegmatis	0	0	0	0	1	1	1	0	0	0
M. species	0	5	0	0	7	9	2	3	4	2
M. szulgai	6	3	3	4	5	1	8	6	6	7
M. terrae cpx	7	3	2	4	4	5	8	6	5	2
M. thermoresistibile	0	0	0	0	0	1	1	0	0	0
M. timonense	0	0	0	0	0	0	0	0	0	6
M. triplex	0	0	1	5	0	0	1	1	0	0
M. triviale	0	0	0	0	0	0	1	0	2	0
M. wolinskyi-jacuzzii	0	0	0	0	1	0	0	0	0	1
M. xenopi	126	127	138	132	88	124	156	102	133	127
M. gordonae + M. xenopi	0	0	1	1	1	0	4	2	0	1
M. chelonae + M. intracellulare cpx	0	0	0	0	0	0	0	0	0	1
M.abscessus + M. avium	0	0	0	0	0	0	0	1	0	0

M. avium + M. intracellulare cpx	0	0	0	1	1	0	0	2	0	1
M. fortuitum + M. intracellulare cpx	0	0	0	0	0	0	1	0	0	0
M. intracellulare cpx + M. xenopi	0	0	0	0	0	0	1	0	0	0
M. kansasii + M. xenopi	0	0	0	0	0	0	1	0	0	0
M. avium + M. simiae	0	0	0	0	0	0	2	0	0	0
M. avium + M. xenopi	0	0	0	0	0	1	0	0	0	0
M. avium + M. gordonae	0	0	0	0	1	1	0	0	0	0
M. intracellulare cpx + M. gordonae	0	0	0	1	1	2	0	0	0	2
M. gordonae + M. kansasii	0	0	0	0	0	1	0	0	0	0
M avium + M. fortuitum	0	0	0	0	1	0	0	0	0	0
M. gordonae + M. simiae	0	0	1	0	0	0	0	0	0	0
Uncharacterized/other NTMs	11	2	0	0	0	0	0	0	0	0
Total NTMs	598	564	698	672	696	715	881	857	896	867
M. tuberculosis	415	468	397	491	467	422	505	429	529	541
BK	5	0	0	0	0	0	0	0	0	0
M. bovis	3	8	5	15	12	3	14	13	10	15
M. bovis BCG	3	1	5	8	10	5	10	5	7	11
M. africanum	1	0	0	0	2	2	7	3	5	3
M. tuberculosis + NTM	0	2	2	3	2	7	3	10	1	0
Total MTBc	427	479	409	517	493	439	539	460	552	570
Total true positives	1014	1043	1107	1189	1189	1154	1420	1317	1448	1437

NTM: nontuberculous mycobacteria; MTBc: mycobacteria tuberculosis complex; cpx: complex; We counted as MTBc the samples in which MTBc and NTM were detected.

Table S5: Frequency over time of antibiograms by non-tuberculous mycobacteria species

Cpx: complex

NTM Species	2007	2008	2009	2010	2011	2012	2013	2014	2015	2016
M. avium	58	59	75	60	65	78	82	79	99	104
M. intracellulare	29	38	40	30	48	50	66	58	38	24
M. chimaera	0	0	0	0	0	0	0	0	37	49
M. xenopi	30	32	25	22	18	22	45	23	37	35
M. kansasii	24	36	21	25	17	17	17	16	25	14
M. chelonae-abscessus cpx	0	0	10	10	14	14	19	22	18	23
M. fortuitum cpx	0	0	5	3	4	8	8	5	8	13
M. malmoense	7	6	0	0	7	6	5	6	6	5
M. marinum	5	6	6	5	1	11	8	11	9	6
M. interjectum	3	0	0	0	0	4	0	0	0	0
M. szulgai	2	3	0	0	0	0	0	0	0	0
M. simiae	0	0	2	1	0	4	0	0	0	0
Other	7	7	9	14	8	13	29	29	20	32
TOTAL	**165**	**187**	**193**	**170**	**182**	**227**	**279**	**249**	**297**	**305**
% of total NTM-positive cultures	**28.1**	**33.2**	**27.7**	**25.3**	**26.1**	**31.7**	**31.7**	**29.1**	**33.1**	**35.2**
